# Anti-SARS-CoV-2 spike IgG following injection of the third dose vaccine: A systematic review with meta-analysis of heterologous versus homologous vaccination

**DOI:** 10.3389/fpubh.2022.960598

**Published:** 2023-01-12

**Authors:** Mohammad-Shafi Mojadadi, Seyed Alireza Javadinia, Fahimeh Attarian, Elham Samami, Mona Sobhani

**Affiliations:** ^1^Department of Immunology, School of Medicine, Sabzevar University of Medical Sciences, Sabzevar, Iran; ^2^Leishmaniasis Research Center, Sabzevar University of Medical Sciences, Sabzevar, Iran; ^3^Non-Communicable Diseases Research Center, Sabzevar University of Medical Sciences, Sabzevar, Iran; ^4^Department of Epidemiology and Biostatistics, School of Health, Torbat Heydariyeh University of Medical Sciences, Torbat Heydariyeh, Iran; ^5^University of Florida Health Cancer Center, Gainesville, FL, United States; ^6^College of Nursing, University of Florida, Gainesville, FL, United States; ^7^Student Research Committee, Sabzevar University of Medical Sciences, Sabzevar, Iran

**Keywords:** SARS-CoV-2, COVID-19 vaccine third dose, heterologous vaccination, homologous vaccination, anti-SARS-CoV-2 antibody

## Abstract

**Background:**

The mass vaccination is a key strategy to prevent and control the coronavirus disease 2019 (COVID-19) pandemic. Today, several different types of vaccines against severe acute respiratory syndrome coronavirus 2 (SARS-CoV-2) have been developed worldwide. These vaccines are usually administered in a two-dose schedule, and the third dose is currently being administered in most countries. This study aimed to systematically review and meta-analyze the immunogenicity of heterologous vs. homologous vaccination after administration of the third dose of COVID-19 vaccines.

**Methods:**

Electronic databases and websites including Scopus, PubMed, Web of Science, and Google scholar were searched for relevant randomized clinical trial (RCT) studies. After applying the inclusion and exclusion criteria, a total of three RCTs were included in the study. These RCTs were included 2,613 healthy adults (18 years or older and without a history of laboratory-confirmed COVID-19) with 15 heterologous and five homologous prime-boost vaccination regimens. Anti-SARS-CoV-2-spike IgG levels at day 28 after administration of the third dose, were compared between the heterologous and homologous regimens.

**Results:**

The highest antibody responses had been reported for the homologous vaccination regimen of m1273/m1273/m1273 (Moderna), followed by the heterologous regimen of BNT/BNT/m1273. In addition, the immunogenicity of viral vector and inactivated vaccines was remarkably enhanced when they had been boosted by a heterologous vaccine, especially mRNA vaccines.

**Conclusion:**

This systematic review suggests that mRNA vaccines in a homologous regimen induce strong antibody responses to SARS-CoV-2 compared to other vaccine platforms. In contrast, viral vector and inactivated vaccines show a satisfactory immunogenicity in a heterologous regimen, especially in combination with mRNA vaccines.

## 1. Introduction

In 2019, a new coronavirus strain known as severe acute respiratory syndrome coronavirus 2 (SARS-CoV-2) emerged in China and quickly spread around the world. Coronavirus disease 2019 (COVID-19), the disease caused by SARS-CoV-2, has had substantial detrimental health and economic impacts on different countries. As of March 2022, more than 508 million people have been infected, and more than 6 million people have died due to COVID-19 (1).

Although many efforts have been made to eradicate or control the disease up to now, SARS-CoV-2 spread is still rising in many world regions. One of the strategies that are believed to be effective, at least in controlling and managing the COVID-19 pandemic, is the mass vaccination of world people. Several companies around the world have developed vaccines against SARS-CoV-2 using various platforms (including ribonucleic acid, non-replicating viral vector, whole inactivated virus, and protein subunit), of which 10 have been licensed for emergency use by the World Health Organization (WHO), including BNT162b2, mRNA-1273, ChAdOx1 nCoV-19, Ad26.COV2.S, Covishield, CoronaVac, BBIBP-CorV, Covaxin, NVX-CoV2373, and Novavax (2, 3). These vaccines are usually injected in a two-dose schedule with a minimum interval of 4 weeks (4).

Although the injection of two doses of these vaccines has significantly prevented mortality and hospitalization due to COVID-19 (5, 6), there is evidence of waning immunity over time (7–10). Therefore, to maintain immunity against COVID-19, the injection of a third dose vaccine (booster dose) is being performed in most countries (11, 12). Studies have shown that injection of the third dose of COVID-19 vaccine (whether homologous or heterologous) can significantly increase the level of anti-spike protein IgG, anti-receptor binding domain (RBD), as well as neutralizing antibodies (even against new variants such as delta and omicron) that might be finally resulted in overcoming the COVID-19 pandemic and associated burnout and pressure on healthcare systems (13–15). Serum levels of these antibodies are directly correlated to the protection against COVID-19. In a study by Munro et al., injection of m1273 (Moderna) vaccine to the individuals who had previously received two doses of BNT (Pfizer) or ChAd (AstraZeneca) vaccines could increase anti-spike protein IgG up to 11.5 and 32.3 times, respectively, compared to control group (receiver of MenACWY, quadrivalent meningococcal conjugate vaccine) (14).

At the beginning of COVID-19 vaccination, due to the shortage of vaccine and delay of supply, and also due to a rare but dangerous complication of blood clotting after receiving the first dose of ChAd vaccine, some countries inevitably used a heterologous vaccine in the second dose (16, 17). Interestingly, heterologous vaccination (i.e., administration of different vaccines in prime-boost schedules) not only had no unbearable adverse events in vaccinees but also was more immunogenic than homologous vaccination (i.e., administration of same vaccines in prime-boost schedules) (18–20). This issue has been well reviewed and discussed in three meta-analysis papers published so far (16, 21, 22). However, no systematic review with meta-analysis paper has been yet published about the immunogenicity of heterologous vs. homologous vaccination after injection of the third dose of COVID-19 vaccines. Therefore, in this systematic review study, all articles published as of February 2022 investigating the immunogenicity of heterologous vs. homologous vaccination after injections of the third dose of COVID-19 vaccines have been reviewed and analyzed. In this study, anti-spike IgG level was used as a criterion to compare immunogenicity between the heterologous and homologous vaccination regimens. Of note, in order to directly compare immunogenicity among the studies, we converted, if necessary, anti-spike IgG levels to the international standard unit of binding antibody units (BAU) per milliliter (BAU/mL) by using the conversion factors mentioned in each study.

## 2. Methods

This study is a systematic review and meta-analysis assessing the immunogenicity of heterologous vs. homologous vaccination regimens after the third dose of COVID-19 vaccines in healthy adults (18 years or older and without a history of laboratory-confirmed COVID-19) based on RCT studies published within the last 2 years. This study was conducted under the Guideline of Preferred Reporting Items for Systematic Reviews and Meta-Analyses (PRISMA) checklist (23). The study question was: in the people who received the third dose of COVID-19 vaccines (P), if heterologous vaccine (I) compared with homologous vaccine (C) induces more antibody responses (O).

### 2.1. Search strategy

**Figure 1:**
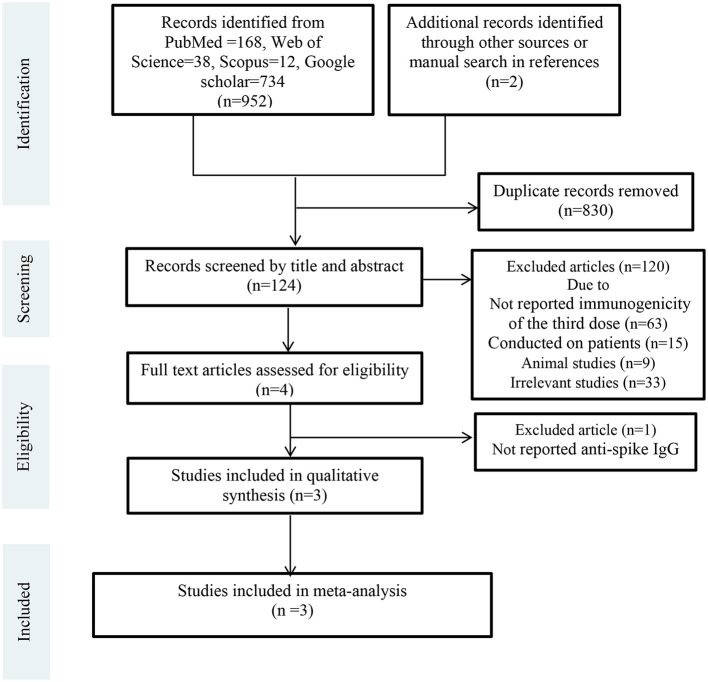
PRISMA flow diagram.

Two separate authors (F.A. and S.A.J.) conducted the online search from electronic databases and websites including Scopus, PubMed, Web of Science, and Google scholar from January 01, 2019, to February 2022. Additionally, we manually screened references or citations of each article. The search terms used in these databases were COVID-19 vaccination, SARS-CoV-2, homologous booster vaccination, heterologous booster vaccination, heterologous prime-boost COVID-19 vaccination, homologous prime-boost COVID-19 vaccination. After the primary search and identification of related studies, we removed duplicate studies. Then, articles were screened by titles and abstracts and irrelevant studies were excluded. Subsequently, full-text versions of the remaining articles (13, 14, 24, 25) were independently assessed for eligibility by two researchers (F.A. and S.A.J.). The third researcher (M.S.M.) monitored the selection accuracy of eligible studies in all steps. These steps are shown in PRISMA Flow Diagram (Figure 1).

### 2.2. Inclusion and exclusion criteria

We included all randomized clinical trials investigating immunogenicity of the third dose of COVID-19 vaccines. The exclusion criteria were as follows: studies conducting on patients, studies with no comparison arm, studies not reporting anti-spike IgG, animal studies, review articles, and editorials.

### 2.3. Quality assessment

The quality of eligible studies was evaluated using the Jadad scale (26). This scale is a good procedure for quality assessment of clinical trials studies. The full text of each study was evaluated by two independent authors (F.A. and S.A.J.). Any disagreements were resolved by discussion with the third author (M.S.M.). Finally, data from unbiased studies entered the meta-analysis (Table 1).

**Table 1:** Quality assessment of studies included to meta-analysis.

	**Atmar et al. (13)**	**Munro et al. (14)**	**Clemens et al. (24)**
Random sequence generation (selection bias)	No	Yes	Yes
Allocation concealment (selection bias)	No	Yes	Yes
Blinding (performance bias and detection bias)	No	Yes	Yes
Incomplete outcome data (attrition bias)	Yes	Yes	Yes
Selective reporting (reporting bias)	No	No	No
Other bias	No	No	No

### 2.4. Data extraction

M.S.M and F.A extracted the data. The concentration of anti-spike IgG at day 28 after injection of the third dose, was selected as a criterion to compare immunogenicity between heterologous vs. homologous COVID-19 vaccination regimens. Of note, to directly compare immunogenicity among the studies, we converted, if necessary, anti-spike IgG levels to the international standard unit of binding antibody units (BAU) per milliliter (BAU/mL) by using the conversion factors mentioned in each study. Before analysis, these data were log-transformed (Log10).

The other main variables that were extracted from the studies were: first author's name, publication year, sample size, mean age and gender of participants, type of vaccination regimens (heterologous or homologous), and type of vaccines [m1273=mRNA-1273 vaccine (Moderna), BNT=BNT162b2 vaccine (Pfizer–BioNTech), Ad26=Ad26.COV2.S (Johnson & Johnson's Janssen), ChAd=ChAdOx1 (Oxford–AstraZeneca), NVX=NVX-CoV2373 (Novavax), SARS-CoV-2 Vaccine Vero Cell (Sinopharm)].

### 2.5. Statistical analysis

Forest plot was created using Comprehensive Meta-Analysis (CMA) software version 3. The forest plot represents the point and overall effect size with 95% confidence interval (CI) of standardized mean differences (SMD) of anti-spike IgG levels between heterologous and homologous vaccination regimens using random effect model. I^2^ statistic was used as a measure of heterogeneity among the studies. *P* < 0.05 was considered statistically significant.

## 3. Results

### 3.1. Study selection and characteristics of the studies included

Totally 954 records were retrieved from electronic databases and websites. After removing duplicates (*n* = 830), 124 studies were screened by title and abstract. Of these, 120 records were excluded due to not reported immunogenicity of the third dose (*n* = 63), conducted on patients (*n* = 15), animal studies (*n* = 9), and other reasons (*n* = 33). Four articles were assessed for eligibility by full-text. One article was excluded because it had not reported anti-spike IgG level. Finally, three RCTs were included in the meta-analysis (13, 14, 24). Figure 1 shows the details of the PRISMA flow diagram.

Totally, 4,876 healthy adults (18 years or older and without a history of laboratory-confirmed COVID-19) had been enrolled in these three trials. Of them, 2,613 had received the third dose of different types of COVID-19 vaccines and their data were included in this meta-analysis. The mean age of participants was 58.2 years, and nearly 53% of them was female (*n* = 1,394). Table 2 shows the main characteristics of the included studies, as well as the serum levels of anti-spike IgG at day 28 after administration of the third dose of COVID-19 vaccine for 15 heterologous and five homologous vaccination regimens.

**Table 2:** Characteristics of the studies included in meta-analysis.

**References**	**Type of primary series vaccines**	**Type of third dose vaccines**	***N***	**Mean/median age (year)**	**Gender (Female) (n)**	**Homologous**	**Heterologous**
						**Anti-spike IgG concentration (BAU/mL) (CI 95%)**	
Munro et al. (14)	BNT/BNT	BNT	96	62.6	61	3,413 (3,025, 3,850)	-
	BNT/BNT	NVX	101	62.1	65	-	1,361 (1,129, 1,641)
	BNT/BNT	chAd	97	61.9	57	-	1,682 (1,466, 1,929)
	BNT/BNT	Ad26	87	62	60	-	2,140 (1,815, 2,522)
	BNT/BNT	m1273	91	63	63	-	4,231 (2,232, 5,136)
	ChAd/ChAd	ChAd	99	63.7	54	308 (258, 367)	-
	ChAd/ChAd	NVX	95	63.5	61	-	874 (730, 1,046)
	ChAd/ChAd	Ad26	98	65	4	-	691 (582, 820)
	ChAd/ChAd	m1273	96	63.8	48	-	3,898 (3,303, 4,600)
	ChAd/ChAd	BNT	93	65.1	50	-	2,571 (2,220, 2,977)
Atmar et al. (13)	Ad26/Ad26	Ad26	50	50	14	369 (291, 476)	-
	Ad26/Ad26	BNT	52	48	14	-	2,277 (1,833, 2,828)
	Ad26/Ad26	m1273	53	57	14	-	2,986 (2,478, 3,598)
	BNT/BNT	BNT	49	50	18	3,164 (2,646, 3,779)	-
	BNT/BNT	Ad26	50	50	15	-	2,600 (2,086, 3,240)
	BNT/BNT	m1273	50	55	17	-	5,231 (4,274, 6,404)
	m1273/m1273	m1273	51	53	16	6,224 (5,282, 7,333)	-
	m1273/m1273	Ad26	49	50	17	-	4,560 (3,544, 5,867)
	m1273/m1273	BNT	51	54	17	-	5,273 (4,567, 6,088)
Clemens et al. (24)	CoronaVac/CoronaVac	CoronaVac	281	58	165	312 (274, 356)	-
	CoronaVac/CoronaVac	Ad26	295	59	181	-	2,173 (1,989, 2,374)
	CoronaVac/CoronaVac	BNT	333	61	204	-	4,349 (3,971, 4,763)
	CoronaVac/ CoronaVac	ChAd	296	60	179	-	2,162 (1,907, 2,452)

### 3.2. Anti-SARS-CoV-2-spike IgG

Three studies had reported anti-spike IgG levels at day 28 following the injection of third dose of different regimens of heterologous and homologous COVID-19 vaccination (13, 14, 24). The results revealed that the highest anti-spike IgG levels belonged to homologous vaccination regimen of m1273/m1273/m1273 (Moderna), followed by heterologous regimen of BNT/BNT/m1273. In addition, the immunogenicity of viral vector and inactivated vaccines was remarkably increased if they had been boosted by a heterologous vaccine, especially mRNA vaccines (Table 2).

**Figure 2:**
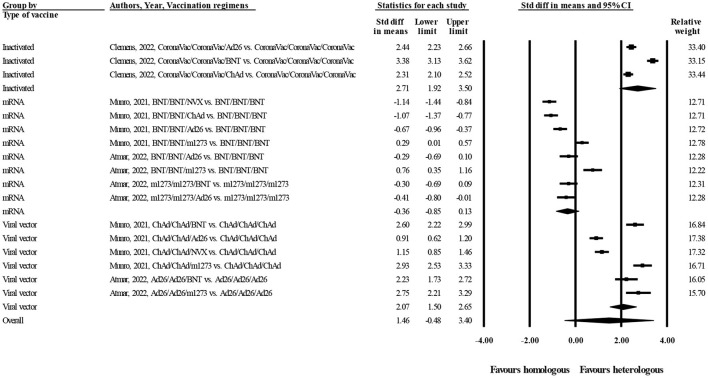
Forest plot of log-transformed concentrations of anti-spike IgG at day 28 after administration of the third dose of COVID-19 vaccines (heterologous vs. homologous vaccination; grouped by type of vaccines).

Figure 2 represents forest plot of standardized mean differences (SMD) of anti-spike IgG concentrations between heterologous and homologous vaccination regimens, grouped by type of vaccines. As shown in this figure, SMD is negative when homologous vaccination regimens belong to mRNA platforms (m1273 and BNT; SMD = −0.36 BAU/mL, 95% CI −0.85–0.13; random effect model, I^2^ = 93%). This means that mRNA vaccines can induce strong antibody responses when administered in homologous regimens. On the other hand, SMD is positive for heterologous regimens of viral vector (ChAd and Ad26, SMD = 2.07 BAU/mL, 95% CI 1.50–2.65; random effect model, I^2^ = 96%) and inactivated vaccines (CoronaVac, SMD = 2.71 BAU/mL, 95% CI 1.92–3.50; random effect model, I^2^ = 96%). This means that to obtain a better immunogenicity, viral vector and inactivated vaccines should be boosted at the third dose by a heterologous vaccine, especially mRNA platforms.

**Figure 3:**
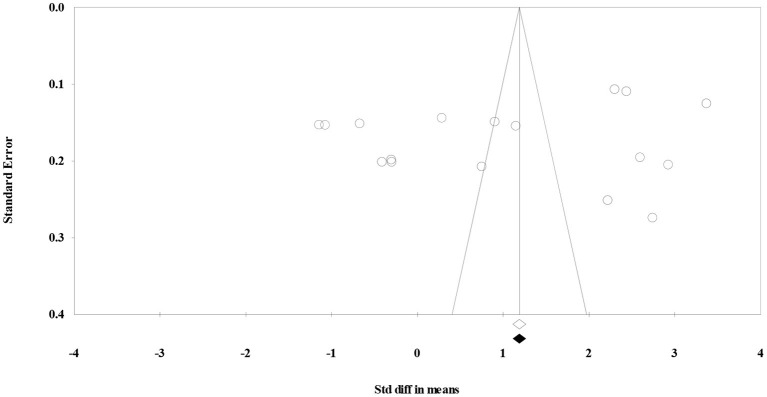
Funnel plot to assess publication bias.

In this study, the publication bias was assessed visually by a funnel plot (Figure 3) and “trim and fill” method (27). The results indicated that under the random effects model the point estimate and 95% CI for the combined studies is 1.05 (0.30–1.80). Using Trim and Fill these values are unchanged. So, there was no publication bias.

## 4. Discussion

In this systematic review, a comprehensive review has been done on the immunogenicity of different types of heterologous and homologous COVID-19 vaccination regimens after injection of the third dose to provide scientific evidence to improve vaccination strategies. To this end, based on the inclusion and exclusion criteria, a total of three randomized clinical trials conducted on 2,613 healthy people (older than 18 years and without a history of laboratory-confirmed COVID-19) were included in the meta-analysis. Our study shows that a significant antibody response against SARS-CoV-2 obtains in a homologous and heterologous vaccination regimen of mRNA vaccines (m1273/m1273/m1273, followed by BNT/BNT/m1273). On the other hand, in case of viral vector and inactivated vaccines, the antibody titers are lower in homologous vaccination regimens compared with heterologous regimens. Interestingly, the immunogenicity of these types of vaccines remarkably enhances when they are administered in a heterologous regimen, especially with a third dose of mRNA vaccines. These findings suggest that mRNA vaccines in a homologous regimen induce strong antibody responses to SARS-CoV-2 compared with other vaccine platforms. In contrast, other vaccine platforms show a satisfactory immunogenicity in a heterologous regimen, especially in combination with mRNA vaccines.

Studies have shown that both humoral and cellular immune responses are important in protecting people from COVID-19 hospitalization and death (28–31). It has been reported that serum levels of anti-spike and anti-RBD antibodies are predictors of immune protection from symptomatic SARS-CoV-2 infection (32, 33), and that neutralizing antibody levels are also correlated to protection from symptomatic infection with SARS-CoV-2 variants of concern, including delta (34) and Omicron (24). Although homologous regimens of a three-dose of mRNA vaccines (m1273 or BNT) generate higher titers of neutralizing antibodies to SARS-CoV-2 D614G pseudovirus (13), delta (B.1.617.2), and omicron variants (B.1.1.529) (24) compared with homologous regimens of adenoviral vectored vaccine (ChAd or Ad26), evidence shows that there is a little difference in initial protection, and server disease or death from SARS-CoV-2 infection after mRNA or adenoviral vector vaccination (28, 35). This may highlight the important role of T cell responses in protective immunity against COVID-19; because viral vector vaccines are somewhat more potent in inducing CD4+ and CD8+ T cell responses against SARS-CoV-2 than mRNA platforms (36). T cell responses also support the generation and maintenance of high-affinity antibodies to SARS-CoV-2.

Based on the discussion mentioned above, it can be concluded that to induce a robust and sustained immunity against SARS-CoV-2, a vaccine should elicit both humoral and cellular immune responses. This may be attained by a heterologous vaccination regimen. For instance, in a study by Atmar et al., it has been reported that injection of an mRNA (m1273 or BNT) vaccine as a third dose to the individuals who had previously received a two-dose of Ad26 platforms, could induce both high titers of neutralizing antibodies and spike-specific Th1 responses in comparison to those receiving a three-dose homologous regimen of Ad26 (13). For this reason, the results of our study should be interpreted with caution, meaning that although a three-dose homologous regimen of mRNA vaccines can induce higher titers of antibody responses than other vaccine platforms, this necessarily does not mean that homologous regimen of mRNA platform is the best choice for COVID-9 vaccination. mRNA vaccines can be a suitable choice as a third dose for those people who have previously received a two-dose of viral vector or inactivated vaccines.

In a similar systematic review and meta-analysis published recently, Cheng et al. have studied the effect of different combinations of homologous and heterologous vaccination regimens on increasing the levels of neutralization and anti-RBD antibodies after the third dose of COVID-19 vaccines (37). In accord with the results of our study, they have reported that the use of mRNA vaccines as a third dose in adults who had previously received two doses of viral vector or inactivated vaccines, can significantly increase antibody responses. In other words, for inactivated and viral vector vaccines, heterologous vaccination regimens are more immunogenic than homologous regimens.

There are some differences between our study and Cheng et al. study (37) that should be mentioned. First, in our study, only RCT studies have been entered into meta-analysis, whereas in Cheng study, RCT as well as observational and non-randomized studies have been included. Second, in our study, anti-spike IgG level has been compared between heterologous and homologous vaccination regimens, whereas in Cheng et al. study, this antibody has not been studied. Third, in Cheng et al. study, all data related to the neutralization and anti-RBD antibodies levels from the original papers, regardless of the measurement day (days 14 or 28 after booster injection) have been entered into meta-analysis, whereas in our study, to control the effect of time variable on the antibody levels, only those studies have been included in the meta-analysis that anti-spike IgG concentrations were measured at day 28 after injection of the third dose. Fourth, in Cheng et al. study, the differences in concentration of neutralization and anti-RBD antibodies before and after injection of third dose of heterologous and homologous vaccination regimens, have been shown in separate forest plots, whereas in our study the differences in anti-spike antibody level among heterologous and homologous vaccination regimens have been shown in one forest plot. This can facilitate transmission of the study message to readers.

There are also some limitations to our study. First of all, there was a substantial heterogeneity (>0.95%) among the studies included that should be taken into account when interpreting the results. The reason for this high heterogeneity may be due to this fact that immune responses to vaccines are affected by many different factors, such as age, sex, race, genetics, lifestyle, nutrition status, body mass index, exercise, and type of vaccine (38). Although in this study, we performed subgroup analysis based on the type of vaccine, however, heterogeneity was still high, probably due to the above-mentioned factors. It seems that this high heterogeneity is inevitable in meta-analysis of vaccine studies, as also seen in Cheng et al. study (37). Second, due to the low number of studies, we could not perform subgroup analysis based on other variables (for example, age, sex, and race). Third, we only searched in English databases, hence the relevant studies that published in other languages may be omitted from our meta-analysis.

## 5. Conclusion

This systematic review suggests that mRNA vaccines in a homologous regimen induce strong antibody responses to SARS-CoV-2 compared to other vaccine platforms. In contrast, viral vector and inactivated vaccine platforms show a satisfactory immunogenicity in a heterologous regimen, especially in combination with mRNA vaccines.

## Data availability statement

The original contributions presented in the study are included in the article/supplementary material, further inquiries can be directed to the corresponding author.

## Ethics statement

The Research Ethics Committee of Sabzevar University of Medical Sciences approved the study protocol (Approval ID: IR.MEDSAB.REC.1401.024).

## Author contributions

M-SM: study concept and design. FA, SJ, and M-SM: acquisition of data. FA: analysis, interpretation of data, and statistical analysis. ES and MS: drafting of the manuscript. M-SM and SJ: critical revision of the manuscript for important intellectual content. All authors contributed to the article and approved the submitted version.
